# Preparation of Crystalline Cefquinome Free Acid via Reverse Anti-Solvent Crystallization: Physicochemical Characterization and Pharmacokinetics in Chickens

**DOI:** 10.3390/pharmaceutics18030333

**Published:** 2026-03-07

**Authors:** Liping Xu, Qiaoyi Zhou, Liangzhu Chen, Feike Zhao, Binghu Fang

**Affiliations:** 1 National Reference Laboratory of Veterinary Drug Residues, College of Veterinary Medicine, South China Agricultural University, Guangzhou 510642, China; 2 Guangdong Provincial Key Laboratory of Tea Plant Resources Innovation and Utilization, Tea Research Institute, Guangdong Academy of Agricultural Sciences, Guangzhou 510640, China; 3 Guangdong Wens Dahuanong Biotechnology Co., Ltd., Yunfu 510610, China

**Keywords:** cefquinome, antisolvent crystallization, solubility, pharmacokinetics, bioavailability, in vitro release study

## Abstract

**Background**: The therapeutic efficiency of cefquinome is currently limited by the low solubility and short half-life of its commercial sulfate form (SCFQ). This study aimed to improve these properties by preparing a novel crystalline cefquinome free acid (CFQ) via a reverse anti-solvent crystallization method. **Methods**: The optimal crystallization conditions were determined through a single factor test. And the product was characterized using X-ray powder diffraction, Fourier transform infrared spectroscopy, and scanning electron microscopy. Meanwhile, in vitro and in vivo pharmaceutical evaluation were conducted. **Results**: CFQ was sucessfully obtained and the optimal crystallization conditions were determined. Comparative in vitro studies showed that CFQ exhibited improved water solubility and dissolution rates compared to SCFQ. In vivo pharmacokinetic evaluations in chickens demonstrated that CFQ significantly prolonged the elimination half-life and increased the area under the concentration-time curve, achieving a relative bioavailability of 139.92%. **Conclusions**: The novel CFQ crystal effectively overcomes the crystallization difficulties of cefquinome and offers a promising alternative formulation with enhanced bioavailability and sustained drug action.

## 1. Introduction

**Figure 1:**
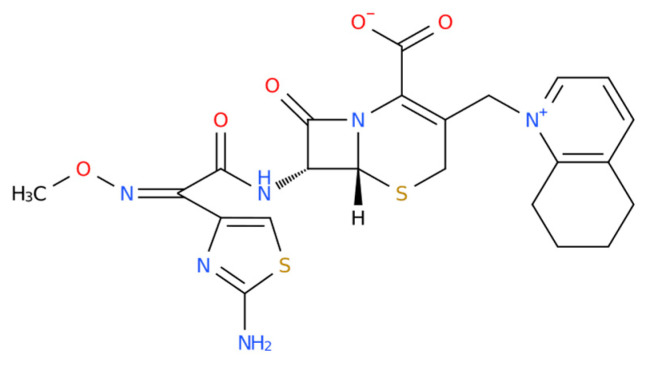
Chemical structure of cefquinome.

Cefquinome is a fourth-generation, animal-specific cephalosporin with broad-spectrum antibacterial activity against both Gram-negative and Gram-positive bacteria [1,2]. The chemical name of cefquinome is (6R, 7R)-7-[[(2Z)-2-(2-amino-1,3-thiazol-4-yl)-2-methoxyiminoacetyl] amino]-8-oxo-3-(5,6,7,8-tetrahydroquinoline-1-ylmethyl)-5-thiadiazabicyclo [4.2.0] oct-2-en-2-carboxylate (Figure 1). The chemical formula is C_23_H_26_N_6_O_5_S_2_ [1,2]. The molecular weight of cefquinome is 528.602 g/mol, with a pH value between 1.5~2.5 [1,2]. And it is widely used for the treatment of respiratory infections in pigs, cattle, as well as poultry pathogen infection [1,2]. In addition, cefquinome exhibits potent activity against important poultry pathogens, including *Pasteurella*, *Escherichia coli*, *Salmonella*, and *Mycoplasma* species [3,4,5,6,7,8]. Owing to its high antibacterial efficacy, low toxicity, and favorable safety profile, with no reported teratogenic, carcinogenic, or mutagenic effects, the development of diverse pharmaceutical formulations of cefquinome has attracted increasing research interest in recent years [9,10,11].

No solid form of cefquinome exists because it is associated with poor stability and a strong tendency toward deliquescence, making storage challenging [12]. Consequently, commercially available cefquinome formulations are primarily marketed as cefquinome sulfate (SCFQ). However, SCFQ still exhibits notable limitations, including low aqueous solubility and a short elimination half-life [2,9]. Current research on novel cefquinome formulations, therefore, focuses on overcoming these drawbacks while exploring combination strategies to achieve synergistic effects [13]. Representative approaches include nanoformulations, freeze-dried powders, and combination formulations. Qu and colleagues developed cefquinome microspheres to prolong the duration of drug action [14], while Du and co-workers prepared micronized SCFQ to enhance permeability and transdermal absorption [15]. Li et al. reported nanogels with gastric acid protection and intestinal targeting capabilities that improved antibacterial activity against *Escherichia coli* [16]. In addition, combining cefquinome with Chinese herbal medicinal ingredients has been shown to enhance antibacterial efficacy and reduce the development of drug resistance [17,18]. Although these strategies improve the performance of SCFQ to varying extents, they often require additional excipients or involve complex manufacturing technologies and specialized equipment.

Altering the solid form of a drug can markedly influence its pharmaceutical performance without the need for additional excipients or complex manufacturing processes. And these different forms can exhibit substantial differences in solubility, dissolution rate, stability, processability, and bioavailability [19]. For example, ceftiofur in the crystalline free acid form shows a longer duration of drug action than ceftiofur sodium [20,21]. However, it is not easy to make cefquinome in the crystalline free acid form. Only an amorphous form could be obtained by many crystalline attempts. Amorphous forms typically possess lower thermodynamic stability and a strong tendency to recrystallize, whereas active pharmaceutical ingredients preferentially exist in more stable crystalline states [22,23]. Accordingly, amorphous cefquinome is inherently unstable and prone to solid-state transformation. Nevertheless, limited attention has been paid to modifying the solid form of cefquinome, such as converting it into a crystalline form. This may largely be attributed to the difficulty of crystallizing cefquinome using conventional crystallization techniques, including solvent evaporation, cooling crystallization, reaction crystallization, and grinding, which have generally failed to induce crystallization of this compound [4,12,24,25,26,27,28].

Previous studies from both domestic and international sources have demonstrated that crystal engineering can effectively improve the physicochemical properties of drugs, enhance solubility, facilitate absorption, and ultimately increase bioavailability [19]. Accordingly, the present study aimed to improve the physicochemical performance of cefquinome by crystallization. A reverse anti-solvent crystallization strategy was employed to prepare cefquinome free acid crystals, thereby overcoming the longstanding challenge of cefquinome crystallization. Compared with conventional crystallization approaches, this method requires lower energy input, operates under milder conditions, and is well-suited for thermally sensitive substances [29]. This study provides experimental support for the development of new crystalline forms of cefquinome and highlights the potential of solid-state transformation as a practical formulation strategy.

## 2. Materials and Methods

### 2.1. Drugs and Chemicals

Cefquinome sulfate (SCFQ; purity 85.1%) was supplied by Aimei Kejian Biomedical Medicine Pharmaceutical Co., Ltd. (Jining, Shandong, China). The SCFQ reference standard (purity 95.0%) was obtained from the Veterinary Drug Administration of China (Beijing, China). Anhydrous sodium carbonate and acetone (analytical grade) were purchased from Guangzhou Guangshi Reagent Technology Co., Ltd. (Guangzhou, China). Acetonitrile, methanol, and anhydrous ethanol (analytical grade) were obtained from Tianjin Fuyu Fine Chemical Co., Ltd. (Tianjin, China). N,N-Dimethylformamide, n-butanol, tetrahydrofuran, and isopropanol (analytical grade) were purchased from Shanghai Runjie Chemical Reagent Co., Ltd. (Shanghai, China). Chromatography-grade acetonitrile was obtained from Thermo Fisher Scientific (Waltham, MA, USA), and chromatography-grade formic acid was purchased from Tianjin Kemio Chemical Reagent Co., Ltd. (Tianjin, China). Ultrapure water was produced in-house using a Milli-Q water purification system (Merck Millipore, Burlington, MA, USA).

### 2.2. Animals

The study was conducted using twenty healthy chickens (10 males and 10 females) with a mean body weight of 2.0 ± 0.2 kg at 135 days of age. The animals were obtained from Qingyuan Fengxiang Chickens Development Co., Ltd. (Qingyuan, China) and housed at the Experimental Animal Center of South China Agricultural University. All chickens were acclimated for at least one week prior to experimentation. During the acclimation period, the animals were subjected to daily clinical examinations to ensure good health and were provided ad libitum access to feed and water free of any medication. Feed was withdrawn 12 h before drug administration, while water remained freely available. The experimental protocol was approved by the Ethical Committee of the Faculty of South China Agricultural University (approval no. 2022A044), and all procedures were performed in accordance with institutional guidelines for the care and use of laboratory animals.

### 2.3. Preparation of CFQ

#### 2.3.1. Preparation of Cefquinome Solution

SCFQ (626.68 g/mol) was added to an aqueous solution of sodium carbonate (105.99 g/mol) in a 1:1 molar ratio. The mixture was continuously stirred until the reaction was complete and a clear solution was obtained, as illustrated in reaction Equation (1). Subsequently, three volumes of acetone were slowly added to precipitate sodium sulfate, which was removed by filtration. A small aliquot of the filtrate was titrated with barium chloride solution (0.01 g/mL) to confirm the complete removal of sulfate ions; the absence of precipitate indicated the completion of sodium sulfate precipitation. The resulting filtrate was a light-yellow cefquinome solution.

(1) $$\mathrm{SCFQ}+Na_{2}{\mathrm{CO}}_{3}\text{→}\mathrm{CFQ}+Na_{2}{\mathrm{SO}}_{4}+{\;\mathrm{CO}}_{2}\text{↑}$$

#### 2.3.2. Crystallization of CFQ

Following the method reported by Wang et al. [12], the cefquinome solution was slowly added to the anti-solvent at 0 °C under a continuous stirring rate of 300 rpm and allowed to crystallize for 8 h. The resulting precipitate was collected by filtration, washed repeatedly with a small volume of anti-solvent, and dried under vacuum to obtain the final product.

#### 2.3.3. Screening of Crystallization Parameters

During the crystallization process, a single-factor experimental design was employed to evaluate the effects of the anti-solvent system (methanol, N,N-dimethylformamide, tetrahydrofuran, isopropanol, n-butanol, anhydrous ethanol, acetone, and acetonitrile), the cefquinome solution-to-anti-solvent volume ratio (1:5, 1:10, 1:15, 1:20, 1:25, and 1:30), temperature (0, 25, 35, and 50 °C), and stirring rate (100, 300, and 600 rpm) on crystal formation.

### 2.4. Characterization Methods

X-ray powder diffraction (XRD) analysis

X-ray powder diffraction was used to characterize CFQ and SCFQ using an Ultima IV diffractometer (Rigaku, Tokyo, Japan). Diffraction data were collected using Cu Kα radiation (λ = 1.54178 Å) with a step size of 0.02°, a scanning range of 5–50° (2θ), and a scan speed of 8 s per step. The instrument was operated at a voltage of 40 kV and a current of 40 mA.

Fourier transform infrared spectroscopy (FTIR) analysis

CFQ and SCFQ samples were individually mixed with potassium bromide (KBr) and ground thoroughly to obtain a homogeneous mixture. Each sample was diluted to 1% (*w*/*w*) with KBr powder, compressed into transparent pellets, and mounted on self-supporting disks. FTIR spectra were recorded using a Vertex 70 Fourier transform infrared spectrometer (Bruker, Karlsruhe, Germany) over a wavenumber range of 4000–400 cm^−1^.

Scanning electron microscopy (SEM) analysis

The particle morphology of CFQ and SCFQ was examined using a scanning electron microscope (EVO MA 15; ZEISS, Oberkochen, Germany). Samples were mounted on aluminum stubs with double-sided conductive carbon tape and sputter-coated with a thin layer of gold prior to observation. Micrographs were acquired at an accelerating voltage of 15 kV.

#### 2.4.1. In Vitro Release Studies

Due to the low stability of CFQ-acetonitrile (In the stability test, CFQ-acetonitrile degraded by more than 10% when stored at room temperature for five days), only CFQ-acetone was evaluated in the in vitro release studies and the subsequent in vivo experiments.

Solubility measurement

Excess amounts of CFQ-acetone and SCFQ were added to 2 mL of ultrapure water to ensure saturation. The resulting suspensions were maintained at room temperature and continuously shaken for 24 h to reach equilibrium. After equilibration, the samples were filtered through a 0.22 µm membrane filter and appropriately diluted prior to analysis. The aqueous solubility of each sample was determined using high-performance liquid chromatography (HPLC).

HPLC analysis was performed on an Agilent 1260 system (Agilent Technologies, Santa Clara, CA, USA) equipped with a ZORBAX XDB-C18 column (4.6 mm × 250 mm, 5 µm; Agilent) and a UV detector set at 270 nm. The mobile phase consisted of 0.16% formic acid in water and acetonitrile (86.5:13.5, *v*/*v*) delivered at a flow rate of 1.0 mL/min. The injection volume was 20.0 μL, and the column was operated at room temperature.

Dissolution rate measurement

According to the Pharmacopoeia of the People’s Republic of China, [30] CFQ-acetone and SCFQ (200 mg each) were compressed into circular tablets with a tablet diameter of 1 cm and individually placed in a rotating basket containing 500 mL of dissolution medium (phosphate buffer, pH 6.8). Dissolution testing was conducted at 37 ± 0.5 °C with a rotation speed of 100 rpm. At predetermined time points (5, 10, 15, 30, 45, 60, 90, and 120 min), 2 mL aliquots were withdrawn and immediately replaced with an equal volume of fresh dissolution medium to maintain sink conditions. Samples were analyzed under the HPLC conditions described above. The amount of drug dissolved at each time point was quantified using the external standard method based on peak area measurements. Dissolution profiles were constructed from experiments performed in triplicate (*n* = 3).

#### 2.4.2. In Vivo Release Studies

Preparation of CFQ and SCFQ suspension injections

A suspension formulation consisting of medium-chain triglycerides as the vehicle, Span 80 as the surfactant, beeswax as the suspending agent, and vitamin E as the antioxidant was developed. This formulation exhibited good compatibility, redispersibility, and syringeability and was therefore used to prepare CFQ and SCFQ suspension injections for subsequent in vivo release studies.

Administration and plasma collection

Twenty healthy chickens were randomly assigned to two groups and administered a single intramuscular dose of CFQ (test group) or SCFQ (control group), respectively. Both groups received the formulations at a recommended clinical dose of 2.0 mg/kg body weight. [6,31,32] Blood samples were collected from the wing vein into heparinized tubes at 0, 5, 10, 15, 30, and 45 min, and at 1, 1.5, 2, 4, 6, 8, 12, and 24 h following intramuscular administration. Plasma was separated by centrifugation at 3000× *g* rpm for 10 min and stored at −20 °C until analysis.

Determination of cefquinome concentration in chicken plasma

For plasma sample preparation, 1 mL of acetonitrile was added to 200 μL of plasma, followed by vortex mixing for 1 min and sonication for 10 min. The mixture was then centrifuged at 10,000× *g* rpm for 10 min. The supernatant was filtered through a 0.45 μm membrane, transferred to a clean tube, evaporated to dryness under a gentle stream of nitrogen, and reconstituted in 500 μL of ultrapure water. After vortex mixing, the solution was filtered through a 0.22 μm membrane prior to analysis.

Cefquinome concentrations were determined by HPLC under isocratic conditions using a mobile phase of 0.16% formic acid in water and acetonitrile (90:10, *v*/*v*) at a flow rate of 0.8 mL/min. Chromatographic separation was achieved on a ZORBAX XDB-C18 column (4.6 mm × 250 mm, 5 μm; Agilent), with the column temperature maintained at 25 °C. Ultraviolet detection was performed at 270 nm, and the injection volume was 30 μL.

#### 2.4.3. Statistical Analysis

XRD and FTIR spectra, plasma cefquinome concentration–time profiles, and dissolution curves were plotted using OriginPro 2022 (OriginLab Corporation, Northampton, MA, USA) and GraphPad Prism v8.0.2 (GraphPad Software, San Diego, CA, USA). Pharmacokinetic analysis of plasma concentration–time data for each chicken was performed using Phoenix WinNonlin v5.2.1 (Pharsight, Mountain View, CA, USA). Non-compartmental analysis was applied to estimate the main pharmacokinetic parameters. Comparisons of pharmacokinetic parameters between the CFQ and SCFQ groups were conducted using SPSS v22.0 (IBM Corp., Armonk, NY, USA). Statistical significance was assessed using the *t*-test. All pharmacokinetic data are presented as mean ± standard deviation (SD).

Relative bioavailability (F) was calculated according to the following Equation (2):

(2) $$F=\frac{{AUC}_{T}\times D_{R}}{{AUC}_{R}\times D_{T}}$$

where AUC_T_ and AUC_R_ represent the areas under the plasma concentration–time curves of CFQ and SCFQ, respectively, and D_T_ and D_R_ denote the administered doses of CFQ and SCFQ.

## 3. Results

### 3.1. Preparation of CFQ

Determination of anti-solvent system

The results showed at Table 1. No precipitate formed in methanol or N,N-dimethylformamide, with the system remaining as an orange-yellow solution. In contrast, orange-yellow, viscous, oil-like amorphous substances were observed when tetrahydrofuran, isopropanol, n-butanol, or anhydrous ethanol was used as the anti-solvent. Granular precipitates were obtained only in acetone and acetonitrile systems.

Determination of anti-solvent ratio

Because granular precipitates were formed exclusively in acetone and acetonitrile systems, these two solvents were selected for further evaluation of the anti-solvent ratio. As shown in Table 2, when the water–acetone ratio exceeded 1:15, granular precipitates were formed, whereas ratios below 1:15 resulted in the formation of oil-like amorphous substances. A similar trend was observed for the water–acetonitrile system; however, the critical ratio for granular precipitation increased to 1:25.

Determination of temperature and stirring rate

As shown in Table 3 and Table 4, crystallization behavior was strongly influenced by temperature and stirring rate. When the temperature exceeded 50 °C or the stirring rate was reduced below 100 rpm, anti-solvent systems that otherwise produced granular precipitates instead yielded oil-like amorphous substances. In contrast, crystallization behavior at 0 °C showed the opposite trend, favoring the formation of granular precipitates.

### 3.2. Characterization of CFQ and SCFQ

XRD analysis

**Figure 2:**
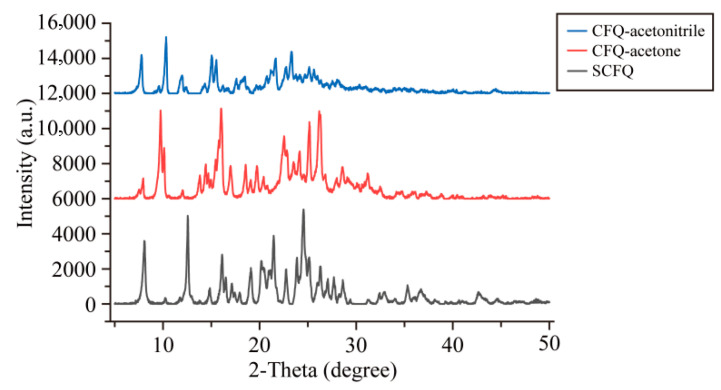
X-ray powder diffraction patterns of CFQ and SCFQ.

The XRD patterns of CFQ-acetone, CFQ-acetonitrile, and SCFQ are shown in Figure 2. CFQ-acetone exhibited characteristic diffraction peaks at 7.999 ± 0.2°, 9.840 ± 0.2°, 10.199 ± 0.2°, 13.979 ± 0.2°, 14.504 ± 0.2°, 16.140 ± 0.2°, 17.100 ± 0.2°, 18.679 ± 0.2°, 19.840 ± 0.2°, 22.620 ± 0.2°, 24.221 ± 0.2°, 25.260 ± 0.2°, and 26.300 ± 0.2°. CFQ-acetonitrile showed distinct diffraction peaks at 7.780 ± 0.2°, 10.320 ± 0.2°, 11.980 ± 0.2°, 15.079 ± 0.2°, 15.520 ± 0.2°, 21.645 ± 0.2°, 22.748 ± 0.2°, 23.326 ± 0.2°, 25.096 ± 0.2°, and 25.609 ± 0.2°. In contrast, SCFQ displayed characteristic peaks at 8.098 ± 0.2°, 12.597 ± 0.2°, 14.846 ± 0.2°, 16.157 ± 0.2°, 19.101 ± 0.2°, 20.275 ± 0.2°, 21.492 ± 0.2°, 22.760 ± 0.2°, 23.950 ± 0.2°, 24.560 ± 0.2°, 25.211 ± 0.2°, 26.320 ± 0.2°, 27.021 ± 0.2°, 27.734 ± 0.2°, and 28.611 ± 0.2°.

FTIR analysis

**Figure 3:**
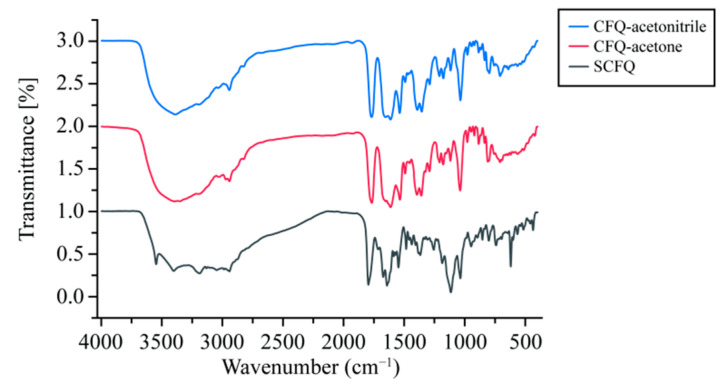
Fourier transform infrared spectra of CFQ and SCFQ.

As shown in Figure 3, the FTIR spectra of CFQ-acetone and CFQ-acetonitrile were highly similar, whereas noticeable differences were observed compared with SCFQ. SCFQ exhibited a stretching vibration band corresponding to the N–H group of –NH_2_ at 3401.93 cm^−1^. A characteristic C=O stretching vibration of the β-lactam ring was observed at 1795.31 cm^−1^, while an absorption band at 1672.49 cm^−1^ was attributed to overlapping vibrations of amide C=O and thiazole ring C=N and C=C bonds. Additional absorption bands associated with the pyridine ring appeared at 3047.30, 1546.77, and 1483.03 cm^−1^. Secondary amide vibrations were observed at 3188.94 and 1641.17 cm^−1^, and methylene and methyl C–H stretching vibrations were detected at 2941.66, 1437.24, and 1367.49 cm^−1^. The stretching vibration of the methyloxime C–O–N group was observed at 1036.00 cm^−1^. In addition, characteristic sulfate-associated bands were present in SCFQ, including an O–H stretching vibration at 3547.20 cm^−1^ and an S=O stretching vibration at 1114.35 cm^−1^. These sulfate-related bands were absent in CFQ-acetone and CFQ-acetonitrile, accompanied by slight shifts in the absorption positions of several functional groups, indicating the removal of sulfate and subtle changes in molecular interactions in CFQ.

SEM analysis

**Figure 4:**
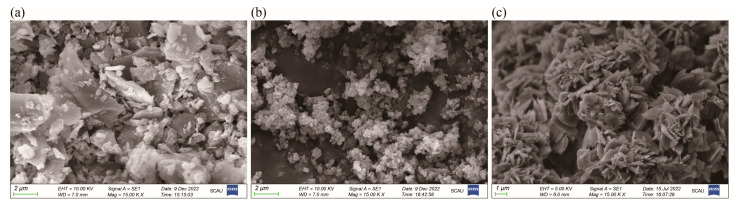
Scanning electron microscopy images of CFQ and SCFQ. Note: (**a**) SCFQ, (**b**) CFQ-acetone, and (**c**) CFQ-acetonitrile.

The surface morphologies of SCFQ, CFQ-acetone, and CFQ-acetonitrile are presented in Figure 4. SCFQ particles exhibited irregular shapes with non-uniform surfaces. In contrast, CFQ-acetone particles appeared predominantly as small, rounded or square-like granules, while CFQ-acetonitrile displayed a flaky morphology.

### 3.3. In Vitro Release Studies

**Figure 5:**
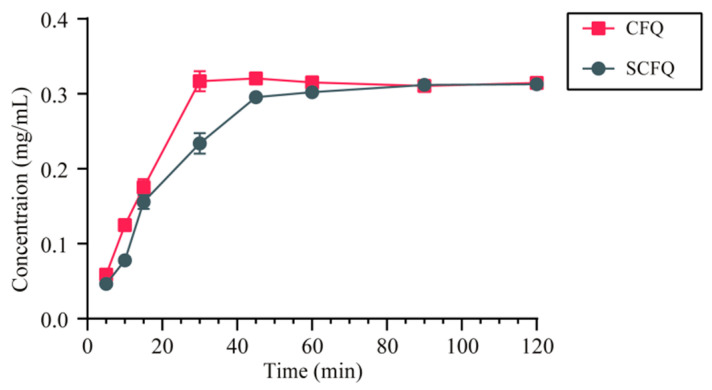
In vitro dissolution profiles of CFQ and SCFQ.

The results showed that CFQ and SCFQ exhibited aqueous solubilities of 74.89 ± 1.27 mg/mL and 31.65 ± 0.13 mg/mL, respectively. The dissolution profiles of CFQ and SCFQ are presented in Figure 5. CFQ achieved dissolution equilibrium within approximately 30 min, whereas SCFQ required approximately 45 min to reach dissolution equilibrium.

### 3.4. In Vivo Release Studies

**Figure 6:**
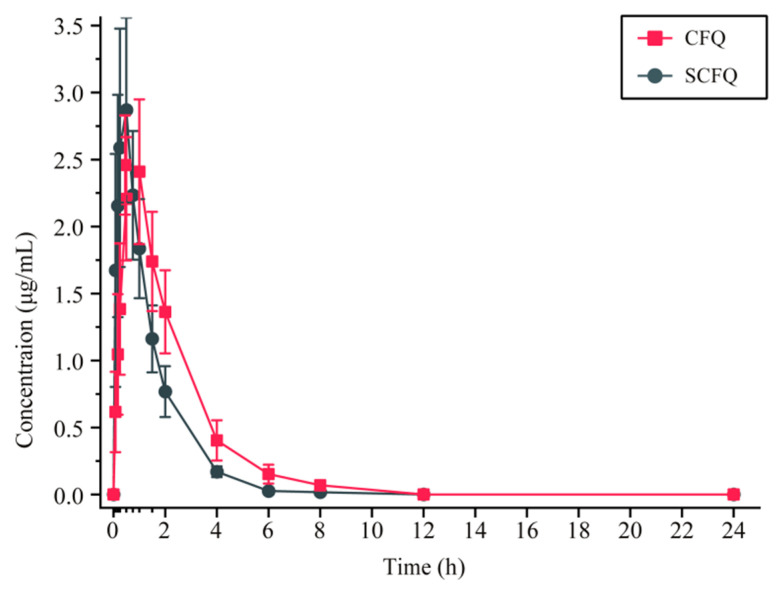
Plasma cefquinome concentration–time profiles in chickens.

Following intramuscular administration of CFQ and SCFQ at a dose of 2 mg/kg in chickens, the main pharmacokinetic parameters are summarized in Table 5, and the corresponding plasma cefquinome concentration–time profiles are shown in Figure 6. For CFQ, the pharmacokinetic parameters were as follows: elimination half-life (t_1/2β_), 1.41 ± 0.28 h; time to peak plasma concentration (T_max_), 0.78 ± 0.22 h; maximum plasma concentration (C_max)_, 2.60 ± 0.47 μg/mL; area under concentration-time curve from zero defining time (AUC_0–last_), 6.19 ± 0.89 μg/mL·h; the clearance (CL/F), 0.32 ± 0.04 L/h/kg; the apparent volume of distribution (V_d_/F), 0.65 ± 0.13 L/kg; and mean residence time to the last measurable concentration (MRT_last_), 1.93 ± 0.23 h. In contrast, SCFQ exhibited a t_1/2β_ of 0.93 ± 0.19 h, a T_max_ of 0.48 ± 0.18 h, a C_max_ of 3.08 ± 0.80 μg/mL, an AUC_0–last_ of 4.43 ± 0.79 μg/mL·h, a CL/F of 0.44 ± 0.08 L/h/kg, a V_d_/F of 0.61 ± 0.23 L/kg, and an MRT_last_ of 1.15 ± 0.09 h. Compared with SCFQ, CFQ showed significantly prolonged t_1/2β_ and T_max_ (*p* < 0.01), a significantly increased AUC_0~last_ (*p* < 0.01), a significantly reduced CL/F (*p* < 0.01), and a significantly prolonged MRT_last_ (*p* < 0.01). The relative bioavailability (F) of CFQ was calculated to be 139.92%.

## 4. Discussion

### 4.1. Preparation of CFQ

At present, a variety of crystallization techniques are available, including solvent evaporation, cooling crystallization, anti-solvent crystallization, grinding, hot-melt extrusion, and supercritical fluid methods [33]. However, only a limited number of these approaches are suitable for the preparation of CFQ. Although hot-melt extrusion offers several advantages, such as the elimination of organic solvents, short processing times, improved conversion efficiency compared with solution-based methods, and compatibility with continuous pharmaceutical manufacturing, it is not appropriate for thermosensitive compounds [34]. In contrast, supercritical fluid techniques are well-suited for thermosensitive substances and can yield solvent-free products with controllable particle sizes, but their application is constrained by high equipment and operational costs [35,36]. Cooling crystallization is generally applicable only to compounds that exhibit substantial solubility changes with temperature [37]. Solvent evaporation crystallization, while broadly applicable, often suffers from prolonged evaporation times that can hinder efficient crystal formation, depending on solvent volatility [34]. Compared with solution-based approaches, grinding is considered a more time-efficient and environmentally friendly method due to the absence of solvents, and it can be conducted at room temperature without temperature dependence [33,38]. Nevertheless, grinding is not widely adopted as a mainstream crystallization technique because it may fail to induce crystallization, result in incomplete conversion, or introduce crystalline defects with residual amorphous content [34].

For the preparation of CFQ, only relatively mild crystallization methods are suitable. During preliminary attempts using grinding, cefquinome readily absorbed moisture from the air, leading to the gradual formation of sticky agglomerates that inhibited crystal formation. In addition, cefquinome is susceptible to degradation under various conditions, including elevated temperature, high humidity, excessive acidity, and excessive alkalinity [39]. Consequently, the use of solvent evaporation crystallization may also result in degradation and loss of drug activity during the prolonged evaporation process. Notably, during CFQ crystallization, a liquid–liquid phase separation phenomenon was observed, characterized by the formation of an orange, viscous, oil-like amorphous phase. This phase separation represents a primary factor leading to crystallization arrest and is a key reason underlying the difficulty of crystallizing cefquinome. Owing to its poor stability and strong tendency toward deliquescence, cefquinome is therefore challenging to convert into a crystalline form using conventional crystallization methods.

Anti-solvent crystallization is well-suited for compounds that are thermally sensitive, prone to degradation, and difficult to crystallize [12]. This approach features a simple process, relatively low equipment requirements, and mild reaction conditions, allowing the entire crystallization procedure to be conducted at room or low temperature [29,33]. Accordingly, anti-solvent methods are widely applied to the crystallization of thermosensitive drugs. In preliminary experiments, CFQ was prepared using conventional (positive) anti-solvent crystallization by adding the anti-solvent dropwise to the cefquinome solution. However, the resulting mild local supersaturation led to a prolonged nucleation induction period [40,41]. The fine crystals that formed were readily encapsulated by the mother liquor or retained solvent on their surfaces, which promoted aggregation and adhesion through solvent-mediated interactions. As a consequence, liquid–liquid phase separation occurred, ultimately resulting in crystallization arrest [42,43,44].

In this study, CFQ was prepared using a reverse anti-solvent crystallization strategy, in which the cefquinome solution was slowly added to the anti-solvent. Under these conditions, cefquinome experienced instantaneous local supersaturation within the anti-solvent, which markedly accelerated the nucleation of small crystal nuclei [12]. As a result, CFQ readily underwent nucleation and precipitation. Moreover, owing to the limited amount of mother solvent present, the interactions between cefquinome molecules and solvent molecules were weakened, thereby reducing the tendency to form a jelly-like phase and favoring crystal formation.

Gao et al. proposed that the occurrence of liquid–liquid phase separation is closely related to the polarity and hydrogen-bonding capability of the anti-solvent [44]. When the hydrogen-bond donor ability of the anti-solvent is stronger, interactions with cefquinome are enhanced, and liquid–liquid separation becomes less likely [12]. Although acetone and acetonitrile possess weaker hydrogen-donating abilities than solvents such as methanol, ethanol, and isopropanol [45], our results demonstrated that CFQ formation occurred only when acetone or acetonitrile was used as the anti-solvent. This behavior may be attributed to the lower solubility of cefquinome in these solvents, which promotes rapid precipitation. In addition, increasing the proportion of anti-solvent was found to facilitate crystal formation. This operation can avoid the formation of a jelly-like phase, which was considered a more serious oiling out phenomenon because of the high viscosity properties [12]. When a sufficiently high proportion of anti-solvent is present, solvent coverage on the crystal surface is reduced, weakening solvent–crystal interactions and thereby suppressing phase separation and aggregation, ultimately favoring crystal formation.

In addition, the effects of temperature and stirring rate on CFQ formation were systematically evaluated. The results demonstrated that both parameters play critical roles in crystallization. When the crystallization temperature exceeded 50 °C, CFQ could not be obtained, even under solvent–anti-solvent ratios (water–acetone, 1:15; water–acetonitrile, 1:25) that successfully yielded CFQ at lower temperatures. In contrast, at 0 °C, CFQ formation was observed at lower anti-solvent ratios (water–acetone, 1:10; water–acetonitrile, 1:20) that failed to produce CFQ at higher temperatures. This behavior may be explained by the exothermic nature of crystallization, whereby reducing temperature thermodynamically favors crystal formation, whereas increasing temperature shifts the equilibrium in the opposite direction and suppresses crystallization [42,46].

Stirring rate was also found to markedly influence crystal formation. When the stirring speed was reduced to 100 rpm, partial transformation of crystals into amorphous material was observed in both acetone and acetonitrile systems. Previous studies have shown that excessively low stirring rates promote crystal aggregation, whereas excessively high stirring rates may disrupt nucleation [47]. Accordingly, an appropriate stirring rate minimizes excessive crystal collisions, reduces aggregation, and limits amorphous phase formation.

Considering the poor stability of CFQ-acetonitrile, the optimal conditions for CFQ preparation were identified as follows: crystallization temperature of 0 °C, acetone as the anti-solvent, a solvent-to-anti-solvent ratio of 1:15, and a stirring rate of 300–600 rpm.

### 4.2. Characterization of CFQ

The XRD results of CFQ-acetone, CFQ-acetonitrile, and SCFQ all exhibited sharp diffraction peaks, confirming their crystalline nature. Compared with SCFQ, CFQ-acetone displayed newly emerged characteristic peaks at 9.840 ± 0.2°, 10.199 ± 0.2°, 13.979 ± 0.2°, 18.679 ± 0.2°, 19.840 ± 0.2°, and 24.221 ± 0.2°, while CFQ-acetonitrile showed distinct peaks at 10.320 ± 0.2°, 15.079 ± 0.2°, 15.520 ± 0.2°, 23.326 ± 0.2°, and 25.609 ± 0.2°. In addition, direct comparison between the two CFQ forms revealed several diffraction peaks unique to CFQ-acetone (9.840 ± 0.2°, 13.979 ± 0.2°, 14.504 ± 0.2°, 16.140 ± 0.2°, 17.100 ± 0.2°, 19.840 ± 0.2°, and 26.300 ± 0.2°) that were absent in CFQ-acetonitrile. Differences in the number and relative intensity of diffraction peaks are characteristic of distinct polymorphic forms and arise from variations in dominant crystal faces and crystallographic plane indices [48]. Although some peak positions were shared among CFQ-acetone, CFQ-acetonitrile, and SCFQ, the observed differences in peak intensity further support differences in crystal structure. These findings indicate that both CFQ-acetone and CFQ-acetonitrile represent new crystalline forms distinct from SCFQ and from each other.

FTIR analysis further demonstrated that CFQ-acetone and CFQ-acetonitrile possessed highly similar spectral features, indicating comparable chemical structures. In contrast, characteristic sulfate-associated bands present in SCFQ, including the O–H stretching vibration at 3547.20 cm^−1^ and the S=O stretching vibration at 1114.35 cm^−1^, were absent in both CFQ forms. In addition, slight shifts in several absorption bands were observed, such as the N–H stretching vibration shifting from 3401.93 to 3389.14 cm^−1^, the β-lactam C=O stretching vibration from 1795.31 to 1766.96 cm^−1^, and the combined C=O/C=N/C=C vibrations from 1672.49 to 1612.12 cm^−1^. These changes indicate the removal of the sulfate group and subtle alterations in intra- or intermolecular hydrogen bonding and molecular packing, while the core chemical structure of cefquinome remained unchanged.

SEM observations revealed clear morphological differences among CFQ-acetone, CFQ-acetonitrile, and SCFQ. CFQ-acetone particles exhibited small, rounded or square-like granular morphologies, CFQ-acetonitrile appeared predominantly flaky, and SCFQ showed irregular particle shapes. Such differences can be attributed to solvent-dependent effects on molecular self-association in supersaturated solutions and on the width of the metastable zone [26]. Variations in solvent–solute interactions influence crystal nucleation, growth, and phase transformation, ultimately leading to differences in molecular packing and crystal form [48].

### 4.3. In Vitro Release Studies

Only CFQ-acetone was evaluated in the in vitro and in vivo release studies due to the low stability of CFQ-acetonitrile. Compared with SCFQ, CFQ exhibited a higher aqueous solubility (74.89 ± 1.27 mg/mL), representing a 236.62% increase and more than a twofold improvement over SCFQ (31.65 ± 0.13 mg/mL). In addition, CFQ demonstrated a faster dissolution rate, achieving dissolution equilibrium within approximately 30 min, whereas SCFQ required approximately 45 min. In addition, we found that the solubility of CFQ in water was higher than in phosphate buffer. Since CFQ is an organic acid, its solubility varies with the pH and composition of the dissolution medium.

In general, solubility enhancement of parent drugs is commonly achieved by salt formation [49]. However, cefquinome represents an exception to this paradigm, as the free acid crystal exhibited superior solubility in water compared with the sulfate salt. This behavior can be attributed to differences in crystal form between CFQ and SCFQ, which result in distinct molecular packing arrangements and intermolecular interactions. CFQ may possess a looser crystal structure with weaker intermolecular forces, facilitating solvent penetration into the crystal lattice and promoting dissolution. Moreover, in the result of SEM, CFQ presented small, rounded or square-like granules while SCFQ presented irregular shapes with non-uniform surfaces. As the SEM result showed that CFQ particles exhibited a smaller size, and it may have a larger effective surface area in contact with the solvent. In addition, CFQ exists as an internal ammonium salt, and its zwitterionic species can directly form stable hydrogen bonds and electrostatic interactions with hydroxide ions in aqueous media, thereby accelerating dissolution [1]. Collectively, these factors account for the superior solubility and dissolution behavior of CFQ relative to SCFQ.

### 4.4. In Vivo Release Studies

The in vivo release results were consistent with those reported by Xie et al. in a pharmacokinetic study conducted in chickens [6]. In the present study, CFQ exhibited a longer t_1/2β_ (1.41 ± 0.28 h) and T_max_ (0.78 ± 0.22 h) than SCFQ (0.93 ± 0.19 h and 0.48 ± 0.18 h, respectively). In addition, AUC_0~last_ increased significantly from 4.43 ± 0.79 µg/mL·h for SCFQ to 6.19 ± 0.89 µg/mL·h for CFQ (*p* < 0.01). The MRT_last was prolonged from 1.15 ± 0.09 h to 1.93 ± 0.23 h, and CL/F decreased from 0.44 ± 0.08 to 0.32 ± 0.04 L/h/kg. As a result, the relative bioavailability of CFQ reached 139.92%. These findings indicate that, at the same administered dose, CFQ undergoes slower absorption and elimination than SCFQ, resulting in a prolonged duration of action and enhanced in vivo drug exposure.

Interestingly, a similar phenomenon has been reported for cefotaxime, where free acid crystals exhibit slower absorption and elimination than cefotaxime sodium [20]. However, in our in vitro release experiment of this study, CFQ has higher water solubility and dissolution rate. In vivo, it needs to transfer from the oil phase to the aqueous phase. Cefquinome is an organic acid with pronounced hydrophilicity and low lipophilicity [32]. As hydrophilicity increases, lipophilicity decreases, which may promote precipitation of CFQ at the intramuscular injection site. The formation of such precipitates could act as a depot, thereby slowing absorption and elimination in vivo. In addition, once CFQ completes its transfer from the oil phase to the aqueous phase, it dissolves efficiently in the tissue fluid due to its higher aqueous solubility, leading to higher bioavailability. Therefore, CFQ exhibits prolonged systemic exposure and higher bioavailability following intramuscular administration.

## 5. Conclusions

At present, commercially available cefquinome is predominantly formulated as SCFQ, which is limited by low aqueous solubility and a short elimination half-life. Although cefquinome itself exists in an amorphous form with relatively higher solubility, this form exhibits poor stability, a strong tendency toward deliquescence, and significant storage challenges. In this study, CFQ was successfully prepared using a reverse anti-solvent crystallization strategy, overcoming the longstanding difficulty associated with cefquinome crystallization. Key process parameters, including anti-solvent type, anti-solvent ratio, temperature, and stirring rate, were systematically optimized using a single-factor experimental approach. The optimal conditions for CFQ formation were identified as a crystallization temperature of 0 °C, acetone as the anti-solvent, a solvent-to-anti-solvent ratio of 1:15, and a stirring rate of 300–600 rpm.

The formation of CFQ was confirmed by XRD, FTIR, and SEM analyses. In vitro studies demonstrated that CFQ exhibited higher water solubility and a faster dissolution rate than SCFQ. In vivo pharmacokinetic evaluation further revealed that CFQ prolonged the duration of drug action, showed slower elimination, and achieved higher bioavailability in chickens (139.92%). Collectively, these findings indicate that solid-state transformation to CFQ effectively enhances the pharmaceutical performance of cefquinome without the need for additional excipients. CFQ, therefore, represents a promising new veterinary formulation with considerable potential for practical application.

## Figures and Tables

**Table 1:** Results of anti-solvent system screening.

Temperature °C	Speed/rpm	Anti-Solvent System	Anti-Solvent Ratio	Result
25	300	Water-methanol	1:5~1:30	S
		Water-N,N-dimethylformamide	1:5~1:30	S
		Water-tetrahydrofuran	1:5~1:30	A
		Water-isopropanol	1:5~1:30	A
		Water-n-butanol	1:5~1:30	A
		Water-anhydrous ethano	1:5~1:30	A
		Water-acetone	1:5~1:30	A/C
		water-acetonitrile	1:5~1:30	A/C

Note: S: orange-yellow solution; A: amorphous substance; C: granular precipitate.

**Table 2:** Results of anti-solvent ratio screening.

Temperature °C	Speed/rpm	Anti-Solvent System	Anti-Solvent Ratio	Result	Anti-Solvent System	Anti-Solvent Ratio	Result
25	300	Water- acetone	1:5	A	water- acetonitrile	1:5	A
			1:10	A		1:10	A
			1:15	C		1:15	A
			1:20	C		1:20	A
			1:25	C		1:25	C
			1:30	C		1:30	C

Note: A: amorphous substance; C: granular precipitate.

**Table 3:** Results of temperature screening.

Speed/rpm	Anti-Solvent Ratio	Temperature °C	Result	Anti-Solvent Ratio	Temperature °C	Anti-Solvent Ratio
300	Water- acetone 1:10	0	C	Water- acetonitrile 1:20	0	C
		25	A		25	A
		35	A		35	A
		50	A		50	A
	Water- acetone 1:15	0	C	Water- acetonitrile 1:25	0	C
		25	C		25	C
		35	C		35	C
		50	A		50	A

Note: A: amorphous substance; C: granular precipitate.

**Table 4:** Results of stirring rate screening.

Temperature °C	Anti-Solvent Ratio	Speed/rpm	Result	Anti-Solvent Ratio	Stirring Rate (rpm)	Result
25	Water- acetone 1:10	100	A	Water- acetonitrile 1:20	100	A
		300	A		300	A
		600	A		600	A
	Water- acetone 1:15	100	A	Water- acetonitrile 1:25	100	A
		300	C		300	C
		600	C		600	C

Note: A: amorphous substance; C: granular precipitate.

**Table 5:** Comparison of the main pharmacokinetic parameters of CFQ and SCFQ.

Parameters	Unit	CFQ	SCFQ
		$\bar{X}$ ± SD, (*n* = 10)	$\bar{X}$ ± SD, (*n* = 10)
t_1/2β_	h	1.41 ± 0.28 **	0.93 ± 0.19
T_max_	h	0.78 ± 0.22 **	0.48 ± 0.18
C_max_	µg/mL	2.60 ± 0.47	3.08 ± 0.8
AUC_0~last_	µg/mL⋅h	6.19 ± 0.89 **	4.43 ± 0.79
CL/F	L/h/kg	0.32 ± 0.04 **	0.44 ± 0.08
Vd/F	L/kg	0.65 ± 0.13	0.61 ± 0.23
MRT_last_	h	1.93 ± 0.23 **	1.15 ± 0.09
F	%	139.92	/

Note: ** *p* < 0.01 compared with SCFQ. t_1/2β_: elimination half-life; T_max_: time to peak plasma concentration; C_max_: maximum plasma concentration; AUC_0~last_: area under concentration-time curve from zero defining time; CL/F: the clearance; Vd/F: the apparent volume of distribution; MRT_last_: mean residence time to the last measurable concentration; F: the relative bioavailability.

## Data Availability

The original contributions presented in this study are included in the article. Further inquiries can be directed to the corresponding author.
